# News and Miscellany

**Published:** 1904-09

**Authors:** 


### News and Miscellany.

Swapped Places.—Quarantine Officers McKnight, of Laredo Station, and Florence, of Rockport Station, have exchanged places for the summer.

Dr. D. M. Reagan, of Buda, Texas, died of cancer August 14, ult., aged 72. He was one of the oldest practicers of medicine in Southern Texas.

Dr. Geo. F. Shrady, thirty-eight consecutive years editor of the New York *Medical Record,* has retired from the editorial chair, and is succeeded by "his assistant," says the *Record.* Name not given.

Will Repair Your Electrical Machines.—Static and all electrical medical apparatus put in running order. I am also agent for electrical and X-Ray apparatus. Oliver Brush, 710 Colorado Street, Austin, Texas.

The Names of Dr. S. R. Burroughs, Dr. I. P. Sessions and Judge Yancey Lewis were accidently omitted from the list of officers of the International Congress on Tuberculosis published in our August issue. They are honorary vice presidents.

The New Orleans Polyclinic has issued its 18th Annual announcement, illustrated. Lots of Texas doctors attended last year. Registrar H. C. Smith, Drawer 602, New Orleans, will send you a copy upon request. A postal card will do it.

For Sale.—On account of wife's health (lung trouble), I will sell for cash, or part cash, seven-acre lot with first class new residence, six rooms, good out improvements, fine orchard, good water, a twelve year's location. Collections good. Address: Dr. R. E. Grigg, Ad Hall, Milam County, Texas.

Ann Arbor, Mich., August 20, 1904.

Dear Dr. Daniel: Please find enclosed my obolus for another year. The "Red Back" gets better every month.

Respectfully,

George Dock.

Want.—To buy practice: Cash. Unopposed practice, worth $2000 a year. Also residence; also drug store, in village on or near railroad (25 miles) in Southwest Texas. Good roads and schools, and no preponderance of Bohemian and German. Temperance locality. Address "Business," care *Texas Medical Journal,* Austin.

THE ANNUAL YELLOW FEVER SCARE is on. One death at Brownsville. The mosquito brigade is on hand and fumigation and screens are the order of the day. Doctor, if you have a case of dengue, deal with it as if it were a case of real yellow fever, *for it is* in my deliberate opinion. *Screen* it, isolate it, destroy the dejecta.

---

DR. WALTER LINDLEY, the editor of the *Southern California Practitioner*, has recently been elected Dean of the Medical College of the University of Southern California. This Los Angeles school is now entering its twentieth session. Dr. Lindley was one of the organizers of the school, and is Professor of Gynecology in that institution.

---

EXIT "THE DAILY."—The third experiment of publishing a daily medical journal in New York has failed,—"owing," I suppose, "to circumstances over which (the publishers) had no control," or something like; didn't get subscribers enough, mayhaps. It went up like a sky-rocket and came down like "a thousand o' bricks." Its large corps of *to-be-paid* staff correspondents got left; at least one down this way did.

---

NEW ORLEANS POLYCLINIC.—Eighteenth annual session opens November 7, 1904, and closes May 20, 1905. Physicians will find the Polyclinic an excellent means for posting themselves upon modern progress in all branches of medicine and surgery. The specialties are fully taught, including laboratory and cadaveric work. For further information, address New Orleans Polyclinic, postoffice box 797, New Orleans, La.

---

A WORLD'S FAIR MAP, with colored map of ground and city, has been issued by the Katharmon Chemical Company, of St. Louis, and will be sent free, with their compliments, on request, naming the "Red Back." It is a complete guide book to the Universal Exposition, and will tell you where to go to find everything worth seeing. All the doctors who are going from Texas—about fifty—to the Tuberculosis Congress alone will want a copy.

---

GOOD LOCATION AVAILABLE.—For sale, a large, fine residence, barn and stables, separate office near by. Seven acres of fine land. Price, $3000; half cash, balance to suit. Practice averages $2500 to $3000. Collections 98 per cent. Practice goes with property. Pretty village in a beautiful and rich farming section peopled with German and Bohemian settlers; prompt pay. Nearest competition ten miles. Dr. Zvesper, Route 3, La Grange, Texas.

---

HOW TO KILL A BABY WITH PNEUMONIA.—Crib in far corner of room with canopy over it. Steam kettle; gas stove (leaky tubing). Room at 80° F. Many gas jets burning. Friends in the room, also the pug dog. Chest tightly enveloped in waistcoat poultice. If child's temperature is 105° F. make a poultice thick, hot and tight. Blanket the windows, shut the doors. If these do not do it, give coal-tar antipyretics and wait.—*Nashville Journal of Medicine and Surgery.*

---

The Election for Legislators is near at hand. "Put none but Americans on guard." "Every man is expected to do his duty," or words to that effect. The medical profession is now a factor in politics and they can and will defeat those who vote *special privileges* to the drugless element, and through medieval notions of economy, refuse to support the State Medical Association's efforts in behalf of the public health. The slogan has gone forth: "No fossil need apply."

---

*Dr. F. E. Daniel, Austin, Texas.*

Dear Doctor:—Find enclosed post office money order in payment of subscription.

The influence of the Journal is most salutory, and is doing much to keep the young practitioner in the straight and narrow path of ethical medicine.

With kindest regards and best wishes to yourself and Our Mascot.

Yours very truly,
F. L. Barnes, M. D.
Trinity, Texas.

---

Texas Delegates to the International Congress on Tuberculosis, St. Louis, October 3, 4, and 5, and all other visitors to the World's Fair, will find the great Iron Mountain Route (Missouri-Pacific System), from Texarkana to St. Louis, and the International and Great Northern (Laredo to Texarkana), and the Cotton Belt (Waco to Texarkana) the best, quickest, most direct route. Dining cars and meals to order, and every comfort and luxury. Service unexcelled. Dr. W. B. Outen, St. Louis, is the chief surgeon of the Iron Mountain. Write to H. C. Townsend, G. P. and T. A., St. Louis, for illustrated folder, mentioning "Red Back."

---

The Passing of "The Senator from Lamar."—Doubtless many of the readers of the Journal will recall that Hon. Travis Henderson, late Senator from Lamar, was an active factor in the defeat of Medical and Sanitary legislation last session of the Legislature, and especially in emasculating the Vital Statistics bill. They will weep large tears, therefore, when they learn that a grateful constituency declined to return him to the Senate, or even to accord him a seat in the lower House, for which he was a candidate,—seeing the hopelessness of trying for the Senate. Senator Patteson of Delta county also got left. It is a mere coincidence (?) that these supporters of the drugless cause were relegated to the shades of private life. The fact that the medical men of Texas are fully organized and wide awake couldn't possibly have had anything to do with it; oh no, not at all.

